# Comparative Analysis of Viromes Identified in Multiple Macrofungi

**DOI:** 10.3390/v16040597

**Published:** 2024-04-12

**Authors:** Kang Zhou, Fan Zhang, Yue Deng

**Affiliations:** 1 Anhui Province Key Laboratory of Environmental Hormone and Reproduction, Fuyang Normal University, Fuyang 236037, China; 2 Anhui Province Key Laboratory of Embryo Development and Reproductive Regulation, Fuyang Normal University, Fuyang 236037, China; 3 State Key Laboratory of Agricultural Microbiology, Huazhong Agricultural University, Wuhan 430070, China; zf1026@yeah.net; 4 Hubei Key Laboratory of Plant Pathology, Huazhong Agricultural University, Wuhan 430070, China; 5 Institute of Plant Protection, Sichuan Academy of Agricultural Sciences, Chengdu 610066, China; 6 Key Laboratory of Integrated Pest Management on Crops in Southwest, Ministry of Agriculture and Rural Affairs, Chengdu 610066, China

**Keywords:** macrofungi, virome, viral diversity, ambi-like viruses

## Abstract

Macrofungi play important roles in the soil elemental cycle of terrestrial ecosystems. Fungal viruses are common in filamentous fungi, and some of them can affect the growth and development of hosts. However, the composition and evolution of macrofungal viruses are understudied. In this study, ninety strains of *Trametes versicolor*, *Coprinellus micaceus*, *Amanita strobiliformis*, and *Trametes hirsuta* were collected in China. Four mixed pools were generated by combining equal quantities of total RNA from each strain, according to the fungal species, and then subjected to RNA sequencing. The sequences were assembled, annotated, and then used for phylogenetic analysis. Twenty novel viruses or viral fragments were characterized from the four species of macrofungi. Based on the phylogenetic analysis, most of the viral contigs were classified into ten viral families or orders: *Barnaviridae*, *Benyviridae*, *Botourmiaviridae*, *Deltaflexiviridae*, *Fusariviridae*, *Hypoviridae*, *Totiviridae*, *Mitoviridae*, *Mymonaviridae*, and *Bunyavirales*. Of these, ambi-like viruses with circular genomes were widely distributed among the studied species. Furthermore, the number and overall abundance of viruses in these four species of macrofungi (*Basidiomycota*) were found to be much lower than those in broad-host phytopathogenic fungi (*Ascomycota*: *Sclerotinia sclerotiorum*, and *Botrytis cinerea*). By employing metatranscriptomic analysis in this study, for the first time, we demonstrated the presence of multiple mycoviruses in *Amanita strobiliformis*, *Coprinellus micaceus*, *Trametes hirsute*, and *Trametes versicolor*, significantly contributing to research on mycoviruses in macrofungi.

## 1. Introduction

Despite the fact that macrofungi have been extensively studied in terms of diversity, research on these organisms still presents many gaps, attracting the interest of researchers around the world. Evolutionarily, macrofungi belong to two main phyla, *Ascomycota* and *Basidiomycota*, while ecologically, they can be associated with dead organic matter, plants, and animals [1]. Macrofungal variety is essential not only for ecosystem maintenance, but also for human survival [2]. Many of these organisms are valuable because they are rich in proteins and vitamins and play an essential role in the human diet [3]. In recent decades, a large number of useful substances from higher fungi with important biological functions, such as blood pressure reduction, immunity enhancement, anti-cancer, and anti-HIV properties, and other pharmacological activities, have been isolated, identified, and characterized [4]. Initially, the demand for edible fungi was met by gathering them from the wild; however, with the recognition of their nutritional value, the demand increased, and eventually, wild collection was supplanted by artificial cultivation [5]. In China, there are over 4000 species of wild macrofungi, of which 1020 are edible species and over 480 dangerous ones [6].

Mycoviruses live and reproduce in the cells of filamentous fungi, yeasts, and oomycetes and are found in all major fungal taxa [7]. The frequency of viral infection ranges from a few percentage points to more than 90% in plant-associated fungi [8]. In 1962, fungal viruses that cause La France disease were first identified in *Agaricus bisporus* [9]. Mycoviruses are generally classified according to the genomic structure and shape of the viral particles, and most of them possess RNA genomes of double-stranded (ds) RNA, positive single-stranded (+ss) RNA, or negative single-stranded (−ss) RNA. Recently, a range of single-stranded, circular DNA viruses have been found in fungi, including *Fusarium graminearum*, *Botrytis cinerea*, and *Sclerotinia sclerotiorum* [10,11,12,13]. According to the International Committee on Taxonomy of Viruses (ICTV), mycoviruses with +ssRNA genomes are classified into twelve families: *Alphaflexiviridae*, *Barnaviridae*, *Botourmiaviridae*, *Deltaflexiviridae*, *Endornaviridae*, *Fusariviridae*, *Gammaflexiviridae*, *Hypoviridae*, *Mitoviridae*, *Narnaviridae*, *Hadakaviridae*, and *Yadokariviridae*. Mycoviruses with −ssRNA genomes are classified into one family and two orders, i.e., *Mymonaviridae, and Serpentovirales and Bunyavirales*, respectively. Mycoviruses with dsRNA genomes are grouped into eight families, *Amalgaviridae*, *Chrysoviridae*, *Megabirnaviridae*, *Partitiviridae*, *Polymycoviridae*, *Quadriviridae*, *Spinareoviridae*, and *Totiviridae*, and one genus, *Botybirnavirus*, in the kingdom *Orthornavirae*. Finally, the family *Genomoviridae* was established for mycoviruses with a single-stranded DNA (ssDNA) genome [14].

The development of high-throughput sequencing (HTS) technologies and various viruses (including several novel viral families) have been identified over the last several years [15,16]. For example, spiliviruses are linked to yeast narnaviruses and include RNA-dependent RNA polymerase motifs that are encoded by several positive-sense (+), single-stranded (ss) RNA genomic segments [17]. Furthermore, ambiviruses consist of small, single-stranded, circular RNA genomes (3–5 kb) with two large open reading frames (ORFs) divided by intergenic regions. They undergo rolling-circle replication and encode their own viral RdRps. In general, ambiviruses are distinct infectious RNAs that show hybrid features of viroid-like RNAs and viruses [14,18]. The majority of mycoviral studies are focused on plant-pathogenic fungi because of the potential for using virus-induced hypovirulence to control fungal infections [19,20]. For example, Cryphonectria hypovirus 1 has been used successfully to suppress chestnut blight in Europe [21,22]. Additionally, it was recently discovered that a single-stranded DNA (ssDNA) genome from the *Genomoviridae* family has a lot of potential for treating *Sclerotinia* disease caused by *Sclerotinia sclerotiorum* by reducing its virulence [10,23]. Although some mycoviruses cause hypovirulence in their host fungi, the vast majority of mycoviral infections are thought to be asymptomatic (or cryptic), which is reinforced by the recent discovery of massive numbers of viruses in various plant-pathogenic fungal populations [24]. On the other hand, fungal virome investigations have also indicated that virus-free fungal individuals may be uncommon in nature and that infection with numerous viruses in one fungal strain is widespread in many fungal species [25,26]. Studies on fungal viromes, similar to those on the viromes of other species, have contributed to our understanding of the diversity of viruses. Studies on fungal viromes, similar to those on the viromes of other species, have contributed to our understanding of the diversity of viruses of several important fungi, including *Sclerotinia sclerotiorum* [17], *Fusarium graminearum* [11], and *Botrytis cinerea* [13]. Many new fungal viruses have been reported, with positive single-stranded RNA viruses predominating. However, there are still large gaps in the study of the viral diversity of macrofungi.

In this study, by using high-throughput sequencing, we described the diversity of fungal viruses from ninety fungal strains of four species (*Amanita strobiliformis*, *Coprinellus micaceus*, *Trametes hirsuta*, and *Trametes versicolor*) that were collected from Wuhan, China.

## 2. Materials and Methods

### 2.1. Sample Collection, DNA and RNA Extraction

In total, 90 fungal strains of the species *Amanita strobiliformis* (*n* = 15), *Coprinellus micaceus* (*n* = 40), *Trametes hirsuta* (*n* = 20), and *Trametes versicolor* (*n* = 15) were collected from Wuhan City, Hubei Province, China, in 2021 (for details, see Supplementary Materials Table S1), and isolated, as described previously [25]. The strains were cultured on potato dextrose agar (PDA) and mycelial plugs stored at −80 °C in 20% glycerol. For DNA extraction, the mycelial plugs of each strain were inoculated on a PDA plate (one plug per plate) covered with cellophane for four days, and the mycelium was then collected. The genomic DNA of each strain was extracted by using the cetyltrime-thylammonium bromide (CTAB) method [27]. Mycelium samples weighing 0.2 g were quickly ground into powder in liquid nitrogen and transferred into 2 mL Eppendorf tubes containing 800 μL of CTAB buffer. After having been evenly mixed, the mixture was heated to 65 °C in a water bath for 0.5 h and then supplemented with 400 μL of phenol and 400 μL of chloroform–isoamyl alcohol (24:1) and evenly mixed again. The mixture was centrifuged at 12,891× *g* for 10 min, and the supernatant was transferred to a new tube. This process was repeated once. Then, the supernatant was mixed with 800 μL of chloroform–isoamyl alcohol and centrifuged at 12,891× *g* for 10 min. The final supernatant was transferred to a new tube, and two times the volume of anhydrous ethanol was added to it. After having been evenly mixed, the mixture was stored at −20 °C for 2 h and then centrifuged at 12,891× *g* for 10 min. The supernatant was removed, and the precipitated DNA was then washed with 100 μL of 75% ethanol twice. After having been air-dried, the genomic DNA was dissolved in 50 μL of ddH_2_O and stored at −20 °C. The ITS-DNA (internal transcribed spacer DNA) regions were obtained using PCR amplification [25].

For RNA extraction, 90 strains were grown for 4 days on a cellophane membrane placed over a PDA plate. Following the manufacturer’s recommendations, 0.5 g of mycelial mass from each strain was collected, and RNA was extracted from it by using a Trizol RNA extraction kit (Aidlab, Beijing, China) [28]. Prior to library construction and sequencing, RNA quality was evaluated using an Agilent 2100 Bioanalyzer (Agilent Technologies, Santa Clara, CA, USA) and quantified using a NanoDrop 2000 (Thermo Fisher, Shanghai, China). Before use, the RNA solutions were stored at −80 °C.

### 2.2. High-Throughput Sequencing, Analysis and RT-PCR Confirmation

Four libraries were generated by combining equal quantities of total RNA (2 μg) from each strain by their species and sending them to GENEWIZ (Suzhou, China) for RNA sequencing (Supplementary Materials Table S1). GENEWIZ performed ribosomal RNA depletion (Ribo-Zero rRNA Removal Kit; Illumina, Inc., San Diego, CA, USA), library preparation (~1 μg of RNA; TruSeq RNA Sample Preparation Kit; Illumina, Inc.), and high-throughput sequencing by using the HiSeq X-ten system (Illumina, Inc.). Unqualified reads were filtered out. The assembly of the ~150 bp readings was performed by using SPAdes (v3.6.1) with the “--meta” option being selected and all other parameters being set to their default values [29]. BLASTx was used to identify sequences similar to the virus-related contigs by using Diamond software (v0.8.22) and the National Center for Biotechnology Information’s non-redundant protein database (https://www.ncbi.nlm.nih.gov/) (accessed on 20 December 2023) [30]. The NCBI ORF Finder program (http://www.ncbi.nlm.nih.gov/gorf/gorf.html) (accessed on 23 December 2023) was utilized to identify the ORFs, and the online motif search tool (https://www.genome.jp/tools/motif/) (accessed on 23 December 2023) was utilized to identify the conserved domains. The multiple-sequence alignment of RNA-dependent RNA polymerase proteins (RdRps), methyltransferase, and conserved helicase domains was performed by using Clustal Omega (https://www.ebi.ac.uk/Tools/msa/clustalo/) (accessed on 25 December 2023). Total RNA was used as a template to synthesize first-strand cDNA and a 25 µL PCR reaction mixture was prepared, containing components like 12.5 µL ExTaq Mix (TaKaRa, Wuhan, China), 1 µL cDNA template, 2 µL specific primers (Supplementary Materials Table S3), and 9.5 µL ddH_2_O, as described previously [28,31]. PCR products were visualized in 1.5% agarose gels (Biowest, Hong Kong, China) with GelRed (Saibeike, Beijing, China). The purified PCR amplicons were inserted into the pMD18-T vector and then transformed into Escherichia coli DH5α competent cells. Three positive clones were selected from the transformed competent cells for sequencing analysis (Tianyi Huayu Gene Technology, Wuhan, China) [31].

### 2.3. Phylogenetic Analysis

The phylogenetic analysis was constructed based on the amino acid sequence of the RdRp region between viral contigs and reference viruses (viral reference sequence selection: a. Similar viral sequences or families were chosen based on the available literature [13,17]; b. NCBI-blastp was used to obtain the sequences that were the closest to the viral contigs in this paper; c. The model species of mycoviruses are downloaded from ICTV (International Committee on Taxonomy of Viruses) (https://ictv.global/taxonomy) (accessed on 25 December 2023), which is related to viral contigs in this work. The Muscle program in MEGA X software (version: 10.1.7) was used for the multiple-sequence alignment of RNA-dependent RNA polymerase proteins (RdRps) and polyproteins. All phylogenetic trees were built by using the neighbor-joining approach with a bootstrap value of 1000 iterations with MEGAX [32]. The sequences of the closest species were used as an outgroup. Some assembly contigs (AsALV3, CmHV1, CmTV1, and CmDFV1, which was too short) did not contain conserved RdRp motifs and could not be used for phylogenetic analysis, so the relationships were analyzed based on the BLASTn results [17].

### 2.4. Viral Diversity

Salmon (v1.9.0) software was used to map the sequence reads to their viral contigs [33]. The viral relative abundance was then calculated as the proportion of viral reads divided by the total reads in the library [34,35]. The richness index was calculated by using the vegan (v2.6-2) package in R [36]. The *t*-test was performed by using the stats (v4.3.1), package in package R (v4.1.0), and *p* < 0.05 was considered to indicate statistical significance. The all-data visualization was performed by using ggplot2 (v3.3.6) software. The raw sequence reads from the meta-transcriptomic libraries are available at the NCBI Sequence Read Archive (SRA) database under the bio-projects PRJNA1060783 (four species of macrofungi), PRJNA632510 (*Botrytis cinerea*), PRJNA778037 (*Botrytis cinerea*), and PRJNA598316 (*Sclerotinia sclerotiorum*). The viral diversity analysis methods for *Botrytis cinerea* and *Sclerotinia sclerotiorum* are consistent with those for macrofungi. Lastly, the assembled contig sequences in this research study were deposited in GenBank databases and are available under the accession numbers indicated in Table 1.

## 3. Results

### 3.1. Metatranscriptomic Identification of Mycoviruses in Four Species of Macrofungi

A total of ninety fungal strains of *Amanita strobiliformis* (*n* = 15), *Coprinellus micaceus* (*n* = 40), *Trametes hirsuta* (*n* = 20), and *Trametes versicolor* (*n* = 15) were identified by sequencing the PCR products of their ITS region. Then, their total RNA was used to construct four meta-transcriptomic sequencing libraries based on the fungal species (the fungal composition of each of the four total RNA pools is available in Supplementary Materials Table S1). The four pools yielded a total of 63 GB of reads, with an average of about 15 GB of reads per pool. After trimming and decontamination, the cleaned reads of each pool were assembled. The contigs from each library were analyzed separately by using BLASTx against a nonredundant protein database, and a total of 20 putative mycoviruses with nearly complete genomes (*n* = 5, containing complete ORFs) or partial contigs (*n* = 15) were identified. They are considered novel mycoviruses or viral contigs, since they share less than 60% amino acid identity with the closest matching reference sequences (Table 1). The e-values, query cover for viral annotation, and viral reads were calculated. The viral contigs were also confirmed by using RT-PCR, and the results show that many strains could be co-infected by multiple viruses (Figure S1; Supplementary Materials Table S2). All the raw sequencing reads were stored in the Sequence Read Archive (SRA) database: BioProject accession No. PRJNA1060783, BioSample accession numbers from SAMN39245089 to SAMN39245092, and SRA runs from SRR27412070 to SRR27412073.

**Figure 1:**
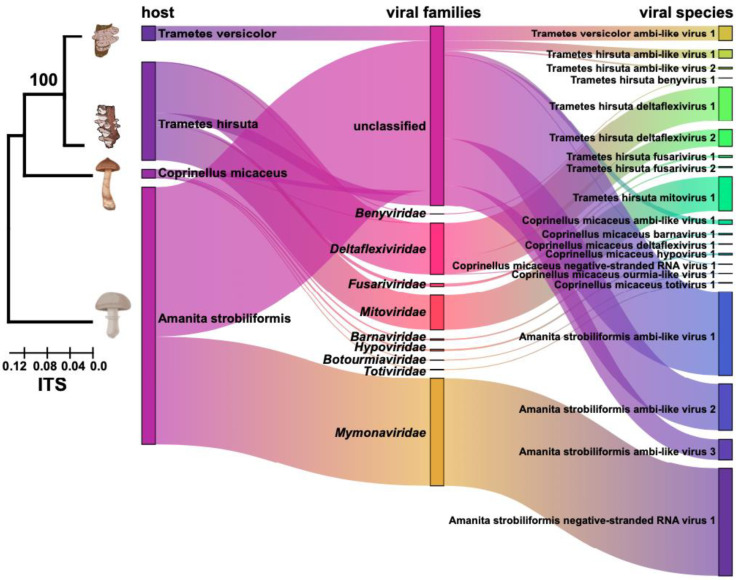
Sankey diagram displaying the compositions of mycovirome from four species of macrofungi. A phylogenetic tree was constructed using ITS sequences based on the neighbour-joining method. The different colors of the Sankey diagram represent the hosts, viral families and species, respectively. The length of the bars represents the viral abundance.

The composition and taxonomy of all mycoviruses are summarized in the Sankey diagram in Figure 1. The putative viral genomes showed affinity with nine distinct lineages, including *Barnaviridae*, *Benyviridae*, *Botourmiaviridae*, *Deltaflexiviridae*, *Fusariviridae*, *Hypoviridae*, *Totiviridae*, *Mitoviridae*, and *Mymonaviridae* (Figure 1). Of the putative viral sequences, the majority (85%) were predicted to represent single-stranded, positive-sense RNA [ss (+) RNA] genomes, and 41% were related to ambi-like viruses (Figure 1). Of the remaining sequences, one was predicted to represent the toti-like virus, and two were predicted to have negative-sense RNA (nsRNA) genomes (Figure 1 and Table 1). The viral compositions were different among the four libraries. The list of putative mycoviruses was summarized for each library, and we found that eight (belonging to the known families *Deltaflexiviridae*, *Mitoviridae*, *Fusariviridae*, and *Benyviridae*) of these viruses originated from *Trametes hirsuta*, seven (belonging to the known families *Hypoviridae*, *Barnaviridae*, *Totiviridae*, *Deltaflexiviridae*, and *Botourmiaviridae*) from *Coprinellus micaceus*, four from *Amanita strobiliformis*, and one from *Trametes versicolor* (Figure 1). None of the viruses were found in more than one species of the analyzed fungi, even though these fungal strains were collected from the same region or have close genetic relationships. In addition, the number of reads on the mapping-to-viral contigs was counted for these viruses (Supplementary Materials Table S2). The relative abundance rates of all mycoviruses identified in the different libraries were calculated. The mycoviruses related with ambi-like viruses were the most abundant, and their reads accounted for more than 47% of all viral reads. At the species level, Amanita strobiliformis negative-stranded RNA virus 1 showed the highest relative abundance, accounting for 29% of all viral reads (Figure 1).

### 3.2. Positive, Single-Stranded RNA Mycoviruses

A total of 17 mycoviruses with +ssRNA genomes were identified in this study. They were classified as *Mitoviridae* (*n* = 1), *Botourmiaviridae* (*n* = 1), *Benyviridae* (*n* = 1), *Barnaviridae* (*n* = 1), *Deltaflexiviridae* (*n* = 3), *Fusariviridae* (*n* = 2), *Hypoviridae* (*n* = 1), and ambi-like viruses (*n* = 7).

**Figure 2:**
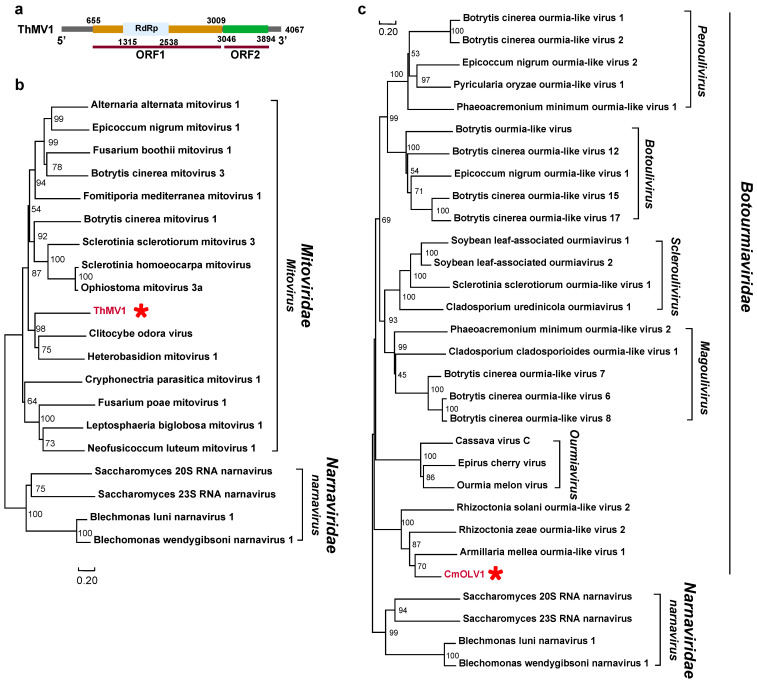
Phylogenetic analysis of mycoviruses belonging to the families *Mitoviridae* and *Botourmiaviridae* and the genome structure of ThMV1. (**a**) The genome structure of ThMV1. (**b**,**c**) The phylogenetic trees of virus sequences within the families *Mitoviridae* and *Botourmiaviridae*, respectively. All amino acid sequences of RNA-dependent RNA polymerases (RdRps) were aligned with Muscle, and then phylogeny was derived using neighbor-joining in MEGA X (bootstrap analysis of 1000 replicates). The viral sequences found in our work were indicated by the red star and the color red.

*Mitoviridae*: A novel mitovirus, named Trametes hirsuta mitovirus 1 (ThMV1), was characterized with two predicted ORFs and has an almost complete sequence of 4067 nt with 43% shared amino acid identity with Lentinula edodes mitovirus 1 (Figure 2a; Table 1). It was identified as belonging to the genus *Mitovirus* based on the phylogenetical tree (Figure 2b).

*Botourmiaviridae*: A viral contig, Coprinellus micaceus ourmia-like virus 1 (CmOLV1), was found to have a partial sequence of 784 nt and 45% shared RdRp identity with members of the family *Botourmiaviridae* (Figure S2; Table 1). The phylogenetic analysis results suggest that CmOLV1 clusters into a group with members belonging to the family *Botourmiaviridae* (Figure 2c).

**Figure 3:**
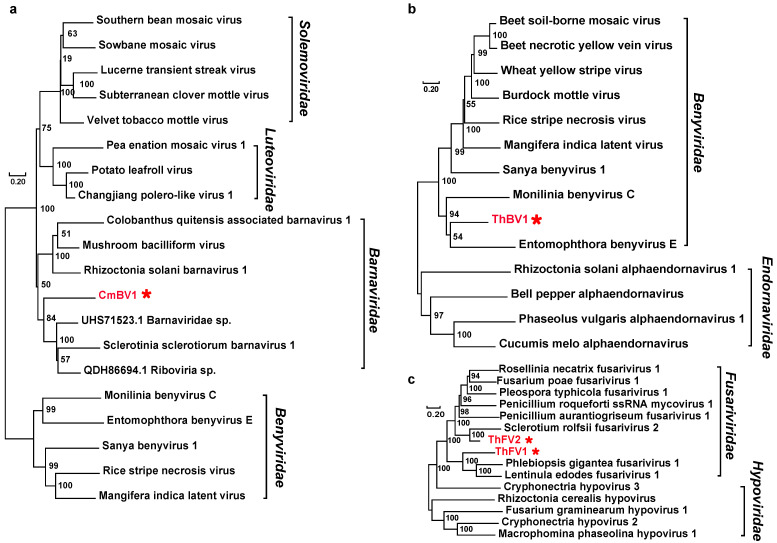
Phylogenetic trees of viral sequences belonged to the families *Barnaviridae*, *Benyviridae*, and *Fusariviridae*, respectively. (**a**) Phylogenetic tree of virus sequences within the family *Barnaviridae*. (**b**) Phylogenetic tree of virus sequences within the family *Benyviridae*. (**c**) Phylogenetic tree of virus sequences within the family *Fusariviridae*. All amino acid sequences of RNA-dependent RNA polymerases (RdRps) were aligned with Muscle, and then phylogeny was derived using neighbor-joining in MEGA X (bootstrap analysis of 1000 replicates). The viral sequences found in our work were indicated by the red star and the color red.

*Barnaviridae*: A 2292 nt incomplete fragment was similar to the barnaviruses and was tentatively named Coprinellus micaceus barnavirus 1 (CmBV1) (Figure S2). The predicted RdRp shares 38% of its identity with Sclerotinia sclerotiorum barnavirus 1, belonging to the family *Barnaviridae* (Table 1). CmBV1 and other barnaviruses are within a well-supported clade of the family *Barnaviridae* (Figure 3a).

*Benyviridae*: One novel viral contig (Trametes hirsuta benyvirus 1; ThBV1) related to the members of the family *Benyviridae* was identified. The partial fragment is 671 nt long, containing the conserved RdRp domain, and shares 42% amino acid identity with Bemisia tabaci beny-like virus 3 (Figure S2; Table 1). The phylogenetic analysis results demonstrate that ThBV1 is grouped with mycoviruses belonging to the family *Benyviridae* (Figure 3b).

*Fusariviridae*: We discovered two novel viruses from the family *Fusariviridae*, tentatively designated as Trametes hirsuta fusarivirus 1 (ThFV1) and Trametes hirsuta fusarivirus 2 (ThFV2). The assembly contigs were 4.95 and 2.23 knt in size, respectively. ThFV1 encodes a polyprotein with conserved RdRp domains and shares 44% identity with the previously identified Phlebiopsis gigantea fusarivirus 1. ThFV2 encodes a polyprotein with conserved RdRp domains and shares 57% identity with the previously discovered Sclerotium rolfsii fusarivirus 2 (Figure S2; Table 1). The phylogenetic analysis results based on the conserved RdRps show that ThFV1, ThFV2, and other members of the family *Fusariviridae* are grouped into one large branch with good support (Figure 3c).

**Figure 4:**
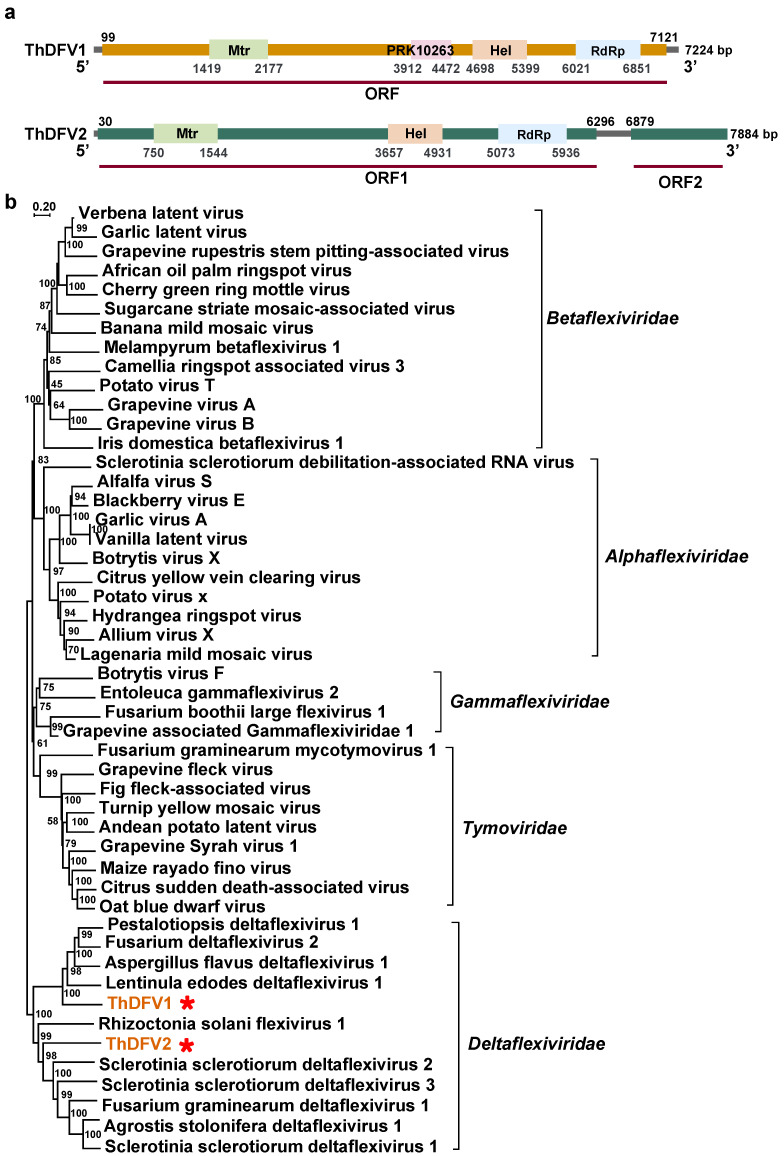
Phylogenetic analysis of sequences belonged to the family *Deltaflexiviridae* and genome structures of ThDFV1 and ThDFV2. (**a**) The genome structures (containing viral methyltransferase, viral helicase, and RdRp) of ThDFV1 and ThDFV2. (**b**) The phylogenetic tree of virus sequences within the family *Deltaflexiviridae*. All amino acid sequences of RNA-dependent RNA polymerases (RdRps) were aligned with Muscle, and then phylogeny was derived using neighbor-joining in MEGA X (bootstrap analysis of 1000 replicates). The viral sequences found in our work were indicated by the red star and the color red.

*Deltaflexiviridae*. We also identified three other mycoviruses belonging to the family *Deltaflexiviridae*, which we tentatively named Trametes hirsuta deltaflexivirus 1 (ThDFV1), Trametes hirsuta deltaflexivirus 2 (ThDFV2), and Coprinellus micaceus deltaflexivirus 1 (CmDFV1). ThDFV1 and ThDFV2 polyproteins (including replicases) share 39% and 32% of their identity with the Cat Tien Macrotermes Deltaflexi-like virus (UUW06602.1) and Lentinula edodes deltaflexivirus 1 (QOX06047.1), respectively (Table 1). The assembled genome of ThDFV1 consists of 7255 nt with one large predicted ORF, which encodes a putative replication-associated polyprotein and contains four conserved domains: methyltransferase, PRK10263, viral helicase 1, and RdRp. ThDFV2 presents an incomplete contig with two ORFs, ORF1 (6267 nt) and ORF2 (>1006 nt), where the former encodes RdRp, viral helicase 1 (Hel), and methyltransferase (Met), respectively, while the latter encodes a protein with an unknown function (Figure 4a). The phylogenetic analysis results show that ThDFV1 and ThDFV2 are in a well-supported clade with other deltaflexiviruses (Figure 4b). The CmDFV1 sequence was incomplete (1273 nt), only a blastp result was obtained, and 47% aa identity was observed between the polyprotein of CmDFV1 and Pestalotiopsis deltaflexivirus 1 (Figure S2; Table 1).

**Figure 5:**
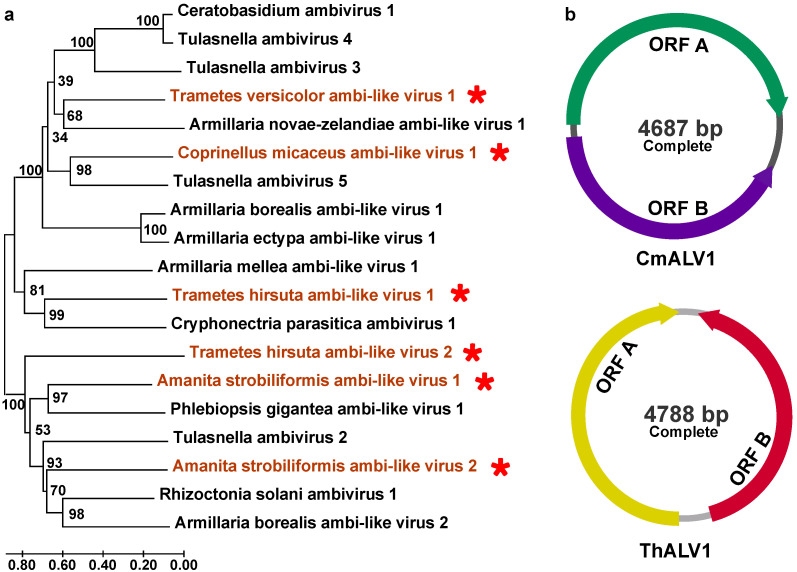
Phylogenetic analysis and genome structure of ambi-like viruses. (**a**) The phylogenetic tree of virus sequences related to ambi-like viruses. All amino acid sequences of RNA-dependent RNA polymerases (RdRps) were aligned with Muscle, and then phylogeny was derived using neighbor-joining in MEGA X (bootstrap analysis of 1000 replicates). (**b**) The genome structures of CmALV1 and ThALV1. The viral sequences found in our work were indicated by the red star and the color red.

Novel +ssRNA viruses with circular genomes: In our investigation, seven putative ambi-like viruses (three from *Amanita strobiliformis*, one from *Coprinellus micaceus*, two from *Trametes hirsute*, and one from *Trametes versicolor*) consisted of circular genomes with two ORFs (Table 1). These were putatively termed Amanita strobiliformis ambi-like virus 1 (AsALV1), AsALV2, AsALV3, Coprinellus micaceus ambi-like virus 1 (CmALV1), Trametes hirsuta ambi-like virus 1 (ThALV1), Trametes hirsuta ambi-like virus 2 (ThALV2), and Trametes versicolor ambi-like virus 1 (TvALV1). The lengths of the obtained fragments ranged from 1258 to 4788 nt (Figure S2). The results of the alignment of the protein sequences with the reference viral sequences suggest that they share amino acid identities ranging from 26% to 40% (Table 1). Among them, the full sequences for ThALV1 and CmALV1 are 4788 and 4687 nt long, respectively. The domain of RNA-dependent RNA polymerase may be encoded by ORFA (Figure 5b). The phylogenetic analysis results demonstrate that they are grouped with the mycoviruses related to ambiviruses (Figure 5a).

### 3.3. Negative Single-Stranded RNA Mycoviruses

**Figure 6:**
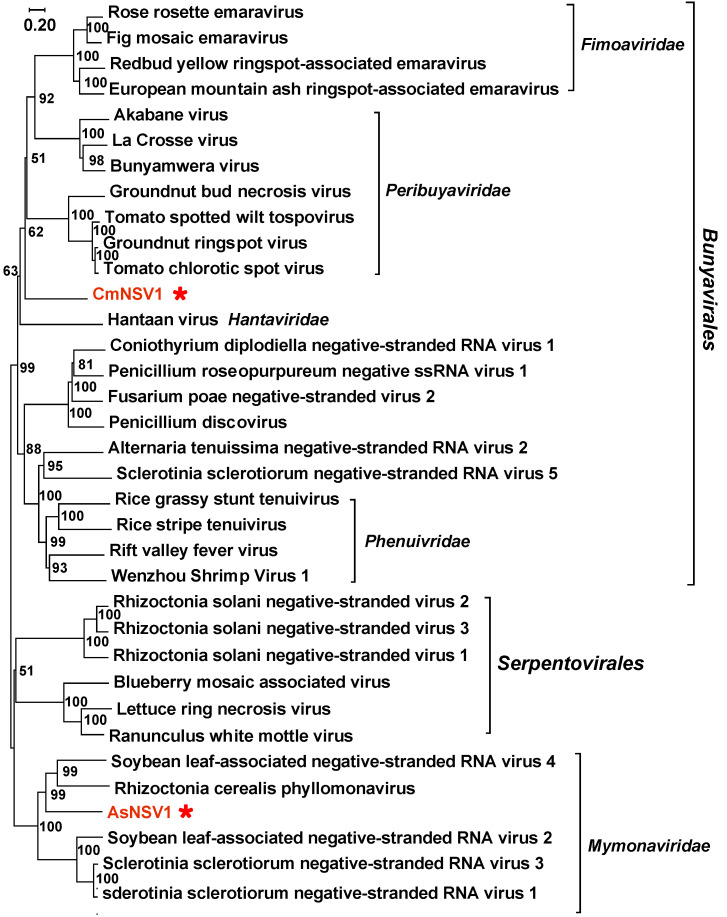
Phylogenetic analysis of putative −ssRNA viruses. All amino acid sequences of RNA-dependent RNA polymerases (RdRps) were aligned with Muscle, and then phylogeny was derived using neighbor-joining in MEGA X (bootstrap analysis of 1000 replicates). The viral sequences found in our work were indicated by the red star and the color red.

*Bunyavirales* and *Mymonaviridae*: One novel viral fragment (Coprinellus micaceus negative-stranded RNA virus 1; CmNSV1) related to the members of *Bunyavirales* was identified. The viral contig is 802 nt, and the partial RdRp encoded by CmNSV1 shares 40% of its identity with that of Phytophthora condilina negative-stranded RNA virus 2 (Table 1). Amanita strobiliformis negative-stranded RNA virus 1 (AsNSV1) was also found to have a partial contig (2421 nt) and to share 34% aa identity with the RdRp of soybean leaf-associated negative-stranded RNA virus 4. A phylogenetic tree was constructed by using the aforementioned RdRp sequences. CmNSV1 forms a well-supported clade with several other fungal bunyaviruses but it is significantly phylogenetically distant from the members of the family *Peribuyaviridae* (Figure 6). AsNSV1 groups with mymonaviruses and could be a new member of the family *Mymonaviridae* (Figure 6).

In addition to the mycoviruses mentioned above, other detected +ssRNA and dsRNA viral contigs included members of the families *Hypoviridae* (*n* = 1) and *Totiviridae* (*n* = 1). Due to incomplete viral sequences (the length of obtained RdRp sequences was not enough for phylogenetic analysis), these viral contigs (AsALV3, CmHV1, CmTV1, and CmDFV1) are only included in Table 1.

### 3.4. Comparison of Viromes from the Phyla Ascomycota and Basidiomycota

**Figure 7:**
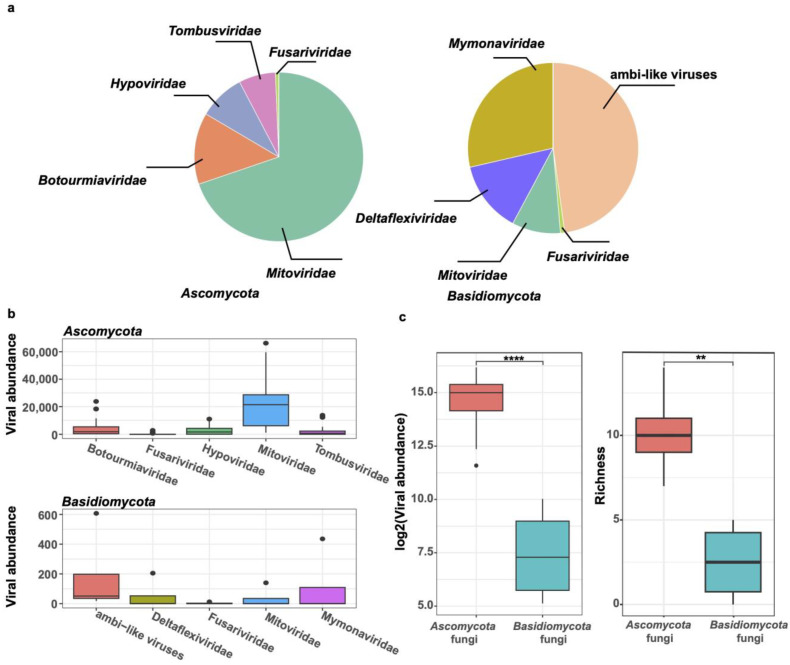
Comparison of mycoviral diversity in the phyla *Ascomycota* and *Basidiomycota* fungi. (**a**) The pie chart shows the proportions of the top five virus families in the phyla *Ascomycota* (*S. sclerotiorum* and *B. cinerea*) and *Basidiomycota* (*A. strobiliformis*, *C. micaceus*, *T. hirsuta*, and *T. versicolor*) fungi. (**b**) The abundance of the top five viral families in two fungal phyla. (**c**) Comparison of the viral abundance and richness index in two fungal phyla (** and ****, representing *p* < 0.01 and 0.0001, respectively).

Previous investigations on mycoviromes have concentrated on important plant-pathogenic fungi. We gathered the mycovirome database from the NCBI-SRA, which includes three viral libraries for Sclerotinia sclerotiorum and thirty viral libraries for Botrytis cinerea, both of which are members of the phylum Ascomycota, and compared it with our findings. The results show that the top five viral families with the highest relative abundance among the fungi of the phylum *Ascomycota* (*S. sclerotiorum* and *B. cinerea*) were *Mitoviridae*, *Botourmiaviridae*, *Hypoviridae*, *Tombusviridae*, and *Fusariviridae*, while the top five viral families among the four species of Basidiomycota fungi were ambi-like viruses, namely, *Mymonaviridae*, *Deltaflexiviridae*, *Mitoviridae*, and *Fusariviridae*, respectively (Figure 7a). The abundance of these viral families varied greatly across the libraries. Moreover, we discovered that at the family level, the total abundance and diversity (richness) of mycoviruses from Ascomycota fungi were much higher than those from Basidiomycota (total viral abundance: *p* < 0.0001; richness: *p* < 0.01) (Figure 7b,c).

## 4. Discussion

Fungi are widely distributed, with conservative estimates ranging from 1.5 to 5 million species on Earth. There are over 2.82 × 10^8^ species of fungal viruses that can parasitize them. However, only a few thousand species of fungal viruses have been characterized [13,17,26,37]. In this study, we showed that various mycoviruses exist in populations of macrofungi (*A. strobiliformis*, *C. micaceus*, *T. hirsute*, and *T. versicolor*) in China. More specifically, twenty putative viral sequences, which share 26% to 57% amino acid identity with known viruses, were identified. According to the published literature, more than 80 distinct viruses with either a dsRNA or ssRNA genome and infecting a total of 34 macrofungal species have been identified. These macrofungal viruses are classified into 12 families (*Partitiviridae*, *Totiviridae*, *Chrysoviridae*, *Endornaviridae*, *Hypoviridae*, *Betaflexiviridae*, *Gammaflexiviridae*, *Barnaviridae*, *Narnaviridae*, *Virgaviridae*, *Benyviridae,* and *Tymoviridae*) [38]. In this study, we obtained many new viral fragments from four species of macrofungi. These viruses could be grouped into ten distinct lineages, including *Barnaviridae*, *Benyviridae*, *Botourmiaviridae*, *Deltaflexiviridae*, *Fusariviridae*, *Hypoviridae*, *Totiviridae*, *Mitoviridae*, *Mymonaviridae*, and *Bunyavirales*. These results imply that macrofungi harbor diverse unknown viruses that are worthy of further exploration.

This is the first report of a mitovirus in *Trametes hirsute*. Mitoviruses are a group of naked viruses with linear +ssRNA genomes, possessing one large ORF that encodes an RdRp of 2.3–3.6 knt in size [39,40,41]. They are thought to replicate in mitochondria, since they often use mitochondrial genetic code for the translation of RdRps [42,43]. In addition, a novel mitovirus, named Heterobasidion mitovirus 3 (HetMV3), was identified in *Heterobasidion annosum*. HetMV3 has a genome length of 5.0 knt and contains three open reading frames (ORFs) [39]. Similarly, a mitovirus, ThMV1, whose nearly complete genome was identified in our study, possesses two ORFs and the six characteristic conserved motifs of mitoviruses [44] (Figure S3). The phylogenetic analysis results also support that ThMV1 belongs to the family *Mitoviridae* (Figure 2b). The amino acid sequence identity was low, 43%, for all reported mitoviruses, so it is thought to be a novel member of the genus *Mitovirus*.

In this study, we determined three novel deltaflexiviruses. The order *Tymovirales* consists of five recognized families (*Alpha*-, *Beta*-, *Delta*-, and *Gamma-flexiviridae*, and *Tymoviridae*) with +ssRNA viral genomes, and all members have a linear genome of 5.9–9.0 kb in length [45]. In this study, only three potential members were identified: ThDFV1, ThDFV2, and CmDFV1. Of these, a BLAST search revealed that ThDFV1 and ThDFV2 are most closely related to Lentinula edodes deltaflexivirus 1 and Cat Tien Macrotermes Deltaflexi-like virus, respectively; their aa sequence identity values are less than 40%. The conserved motifs in the Mtr (methyltransferase), Hel (helicase), and RdRp domains of ThDFV1 and ThDFV2 are similar to those of deltaflexiviruses [46,47] (Figure S4). Furthermore, the phylogenetic analysis results show that they are clustered with viruses in the viral family *Deltaflexiviridae* with 100% bootstrap support (Figure 4b). These results suggest that ThDFV1 and ThDFV2 may be novel members of this family. However, CmDFV1 was not analyzed in depth in this study due to the short sequence obtained.

In this study, we identified seven viral contigs related to ambi-like viruses. The earliest evidence of ambi-like viruses was found in endomycorrhizal fungi [48]. Numerous phytopathogenic fungi, including *C. parasitica* [49], *Armillaria* spp. [50], and *H. parviporum* [51], have also been found to harbor this viral group in recent years. They have a non-segmented RNA genome of 4.5–5.0 kb with an ambisense coding nature that possesses two open reading frames (ORFs) (A and B) on each strand [52]. Moreover, in the above study, the authors also showed that fungal ambi-like viruses contain viroid-like elements that undergo rolling-circle replication and encode their own viral RdRps. Thus, ambiviruses are distinct infectious RNAs showing hybrid features of viroid-like RNAs and viruses [18]. In our study, 7 of 20 were ambi-like viruses, suggesting that these viruses might be widespread in fungi in the phylum *Basidiomycota*, highlighting macrofungi as an evolutionary hub for RNA viruses and viroid-like elements. These findings indicate that an in-depth understanding of virus composition in macrofungi is necessary. However, the effects of these viruses on the host remain unknown and need to be further investigated.

Cross-species transmission of fungal viruses has been reported in other fungi under laboratory conditions, such as *S. sclerotiorum*/*S. minor* [53], *Aspergillus niger*/*A. nidulans* [54], and *C. parasitica/C. nitschkei* [55]. Recent findings have also demonstrated that the cross-species transmission of mycovirus LbBV1 was achieved only through the inoculation of mixed spores of *Leptosphaeria biglobosa* and *B. cinerea* on PDA or on stems of oilseed rape with the efficiency rates of 4.6% and 18.8%, respectively [28]. In all these cases, the donor and recipient species belonged to the same genus or had ecological niche intersections. In our study, the comparison of the viromes of four species of macrofungi showed that the highest number of mycoviruses were identified in *Trametes hirsuta* and the lowest in *Trametes versicolor*. Moreover, none of the viruses were shared among the analyzed macrofungi, implying that viral cross-species transmission among them may rarely occur. The reason for this result may be the different ecological niches or vegetative incompatibility among them.

The composition of fungal viruses in the phyla *Basidiomycota* and *Ascomycota* was quite different. In *Ascomycota* (including *S. sclerotiorum* and *B. cinerea*), we found that viruses belonging to the family *Mitoviridae* appeared to be the dominant viruses, comprising 70% of the total viral reads. However, the dominant viruses among the four species of macrofungi in this study were ambi-like viruses (comprising 47% of the total viral reads). Moreover, the viral richness and total virus abundance detected in the *S. sclerotiorum* and *B. cinerea* samples were significantly higher than those of the four species of macrofungi. In a previous study, it was observed that antiviral RNA silencing was impeded by a mitovirus CpMV1, which is consistent with its mitochondrial replication [56]. Therefore, this may be one reason as to why mitoviruses are widely distributed in *S. sclerotiorum* and *B. cinerea*. On the other hand, viral diversity may be influenced by inter-species horizontal gene transfer events. For example, Sclerotinia sclerotiorum alphaflexivirus 2 was isolated from the hypovirulent strain *S. sclerotiorum*. The phylogenetic analysis results show that SsAFV2 clusters with Botrytis virus X (BVX) based on the multiple-sequence alignment of helicase, RdRp, and CP, but the methyltransferase of SsAFV2 was found to be most closely related to Sclerotinia sclerotiorum alphaflexivirus 1, suggesting that SsAFV2 is a new member of the *Botrexvirus* genus within the *Alphaflexiviridae* family [57]. However, more research is needed to determine why ambi-like viruses are so common among the macrofungi analyzed in further study.

## 5. Conclusions

For the first time, in this study, we showed the existence of various mycoviruses in *Amanita strobiliformis*, *Coprinellus micaceus*, *Trametes hirsuta*, and *Trametes versicolor*. Twenty putative mycoviruses were identified and categorized into ten distinct lineages, including *Barnaviridae*, *Benyviridae*, *Botourmiaviridae*, *Deltaflexiviridae*, *Fusariviridae*, *Hypoviridae*, *Totiviridae*, *Mitoviridae*, *Mymonaviridae*, and *Bunyavirales*. Furthermore, seven viruses or viral contigs with circular genomes, i.e., AsALV1 to AsALV3, TvALV1, CmALV1, ThALV1, and ThALV2, may belong to a novel viral family. In addition, none of the viruses are shared among the analyzed macrofungi, and there is significantly lower mycoviral diversity in these four macrofungi than in other pathogenic fungi (*S. sclerotiorum* and *B. cinerea*). In general, by employing metatranscriptomic analysis, the origin and evolution of many mycoviral groups were studied in macrofungal species.

## Figures and Tables

**Table 1:** Mycoviruses identified in four species of macrofungi.

Viral Name	Length (nt)	Best Match	Identity (aa)	Family	Genome	Accession Number
Amanita strobiliformis ambi-like virus 1	4503	UJT31805.1 Phlebiopsis gigantea ambi-like virus 1	40%	unclassified	+ssRNA	PP105116
Amanita strobiliformis ambi-like virus 2	3116	QMP84024.1 Rhizoctonia solani ambivirus 1	35%	unclassified	+ssRNA	PP105117
Amanita strobiliformis ambi-like virus 3	1258	QPB44667.1 Tulasnella ambivirus 2	26%	unclassified	+ssRNA	PP105118
Amanita strobiliformis negative-stranded RNA virus 1	2421	YP_010784559.1 Soybean leaf-associated negative-stranded RNA virus 4	34%	*Mymonaviridae*	−ssRNA	PP105119
Trametes versicolor ambi-like virus 1	3426	WNH24528.1 Heterobasidion ambi-like virus 15	37%	unclassified	+ssRNA	PP105120
Coprinellus micaceus ambi-like virus 1	4687	QPB44674.1 Tulasnella ambivirus 5	40%	unclassified	+ssRNA	PP105121
Coprinellus micaceus hypovirus 1	3416	WMI40060.1 Rhizoctonia cerealis hypovirus	40%	*Hypoviridae*	+ssRNA	PP105122
Coprinellus micaceus barnavirus 1	2292	UHS71731.1 Sclerotinia sclerotiorum barnavirus 1	36%	*Barnaviridae*	+ssRNA	PP105123
Coprinellus micaceus totivirus 1	1544	WPH57541.1 Rhizoctonia solani toti-like virus 1	48%	*Totiviridae*	dsRNA	PP105124
Coprinellus micaceus deltaflexivirus 1	1273	QTH80200.1 Pestalotiopsis deltaflexivirus 1	47%	*Deltaflexiviridae*	+ssRNA	PP105125
Coprinellus micaceus negative stranded RNA virus 1	802	QTT60994.1 Phytophthora condilina negative stranded RNA virus 2	40%	unclassified	−ssRNA	PP105126
Coprinellus micaceus ourmia-like virus 1	784	YP_010805005.1 Armillaria mellea ourmia-like virus 1	45%	*Botourmiaviridae*	+ssRNA	PP105127
Trametes hirsuta deltaflexivirus 1	7224	QOX06047.1 Lentinula edodes deltaflexivirus 1	39%	*Deltaflexiviridae*	+ssRNA	PP105128
Trametes hirsuta deltaflexivirus 2	7884	UUW06602.1 Cat Tien Macrotermes Deltaflexi-like virus	32%	*Deltaflexiviridae*	+ssRNA	PP105129
Trametes hirsuta ambi-like virus 1	4788	WPS91567.1 Fusarium graminearum ambivirus 1	32%	unclassified	+ssRNA	PP105130
Trametes hirsuta ambi-like virus 2	2980	WNA22195.1 Downy mildew lesion associated ambivirus 2	31%	unclassified	+ssRNA	PP105132
Trametes hirsuta mitovirus 1	4067	QOX06058.1 Lentinula edodes mitovirus 1	43%	*Mitoviridae*	+ssRNA	PP105131
Trametes hirsuta fusarivirus 1	4953	UJT31800.1 Phlebiopsis gigantea fusarivirus 1	44%	*Fusariviridae*	+ssRNA	PP105133
Trametes hirsuta fusarivirus 2	2275	YP_010799383.1 Sclerotium rolfsii fusarivirus 2	57%	*Fusariviridae*	+ssRNA	PP105134
Trametes hirsuta benyvirus 1	671	QWC36503.1 Bemisia tabaci beny-like virus 3	42%	*Benyviridae*	+ssRNA	PP105135

+ssRNA, −ssRNA and dsRNA indicate positive- and negative-strand single-stranded RNAs and double stranded RNAs, respectively.

## Data Availability

The raw sequence reads are available at the NCBI Sequence Read Archive (SRA) database under the bio-project PRJNA1060783, and accession numbers of viral sequences are PP105116-PP105135.

## Supplementary Materials

The following supporting information can be downloaded at: https://www.mdpi.com/article/10.3390/v16040597/s1, Figure S1: PCR confirmation of mycovirus contigs in four species of macrofungi strains (including *Amanita strobiliformis*, *Coprinellus micaceus*, *Trametes hirsuta*, and *Trametes versicolor*). The viral primers were designed according to the contig sequences, and primers used are listed in Supplementary Materials Table S3. Figure S2: A schematic diagram shows the structure of other viral sequences. The conserved domains include RNA-dependent RNA polymerase (RdRp) and viral helicase (Hel), which are indicated on their fragments. Figure S3: Conserved amino acid sequence motifs of the putative RNA-dependent RNA polymerases of Trametes hirsuta mitovirus 1 (ThMV1), Cryphonectria cubensis mitovirus 2a (CcMV2a), Ophiostoma mitovirus 1a (OnuMV1a), Sclerotinia homoeocarpa mitovirus (ShMV), Ophiostoma mitovirus 3a (OnuMV3a), and Cryphonectria parasitica mitovirus 1-NB631 (CpMV1-NB631). “*” indicates identical amino acid residues; and “.” indicates low chemically similar amino acid residues. Figure S4: Multiple alignment of amino acid sequences of conserved domains including methyltransferase (Mtr), viral RNA Helicase (Hel), and RNA-dependent RNA polymerase (RdRp) domains of Trametes hirsuta deltaflexivirus 1 (ThDFV1), ThDFV2, Sclerotinia sclerotiorum deltaflexivirus 1 (SsDFV1), Sclerotinia sclerotiorum deltaflexivirus 2 (SsDFV2), and Fusarium graminearum deltaflexivirus 1 (FgDFV1). “*” indicates identical amino acid residues, and “.” indicates low chemically similar amino acid residues. Table S1: Macrofungi strains from China used in this study. Table S2: The blastp, PCR confirmation and reads of mycovirus contigs in four species of macrofungi. Table S3: Primers used to detect viral contigs.

## References

[1] Tang X., Mi F., Zhang Y., He X., Cao Y., Wang P., Liu C., Yang D., Dong J., Zhang K. (2015). Diversity, population genetics, and evolution of macrofungi associated with animals. Mycology.

[2] Mueller G.M., Schmit J.P., Leacock P.R., Buyck B., Cifuentes J., Desjardin D.E., Halling R.E., Hjortstam K., Iturriaga T., Larsson K.H. (2007). Global diversity and distribution of macrofungi. Biodivers. Conserv..

[3] Chang S., Buswell J. (2023). Medicinal Mushrooms: Past, Present and Future. Adv. Biochem. Eng. Biotechnol..

[4] Xiao H., Zhong J.J. (2016). Production of Useful Terpenoids by Higher-Fungus Cell Factory and Synthetic Biology Approaches. Trends Biotechnol..

[5] Nikšić M., Podgornik B.B., Berovic M. (2023). Farming of Medicinal Mushrooms. Adv. Biochem. Eng. Biotechnol..

[6] Wu F., Zhou L.W., Yang Z.L., Zhu L.B., Li T., Tai H.D., Yu C. (2019). Resource diversity of *Chinese macrofungi*: Edible, medicinal and poisonous species. Fungal Divers..

[7] Ghabrial S.A., Castón J.R., Jiang D., Nibert M.L., Suzuki N. (2015). 50-plus years of fungal viruses. Virology.

[8] Hillman B.I., Annisa A., Suzuki N. (2018). Viruses of Plant-Interacting Fungi. Adv. Virus Res..

[9] Hollings M. (1962). Viruses associated with a die-back disease of cultivated mushroom. Nature.

[10] Yu X., Li B., Fu Y., Jiang D., Ghabrial S.A., Li G., Peng Y., Xie J., Cheng J., Huang J. (2010). A geminivirus-related DNA mycovirus that confers hypovirulence to a plant pathogenic fungus. Proc. Natl. Acad. Sci. USA.

[11] Li P., Wang S., Zhang L., Qiu D., Zhou X., Guo L. (2020). A tripartite ssDNA mycovirus from a plant pathogenic fungus is infectious as cloned DNA and purified virions. Sci. Adv..

[12] Hao F.M., Wu M.D., Li G.Q. (2021). Characterization of a novel genomovirus in the phytopathogenic fungus *Botrytis cinerea*. Virology.

[13] Ruiz-Padilla A., Rodríguez-Romero J., Gómez-Cid I., Pacifico D., Ayllón M.A. (2021). Novel mycoviruses discovered in the mycovirome of a necrotrophic fungus. mBio.

[14] Kondo H., Botella L., Suzuki N. (2022). Mycovirus diversity and evolution revealed/inferred from recent studies. Annu. Rev. Phytopathol..

[15] Ayllón M.A., Vainio E.J. (2023). Mycoviruses as a part of the global virome: Diversity, evolutionary links and lifestyle. Adv. Virus Res..

[16] Rumbou A., Vainio E.J., Büttner C. (2021). Towards the Forest Virome: High-Throughput Sequencing Drastically Expands Our Understanding on Virosphere in Temperate Forest Ecosystems. Microorganisms.

[17] Jia J., Fu Y., Jiang D., Mu F., Cheng J., Lin Y., Li B., Marzano S.L., Xie J. (2021). Interannual dynamics, diversity and evolution of the virome in *Sclerotinia sclerotiorum* from a single crop field. Virus Evol..

[18] Forgia M., Navarro B., Daghino S., Cervera A., Gisel A., Perotto S., Aghayeva D.N., Akinyuwa M.F., Gobbi E., Zheludev I.N. (2023). Hybrids of RNA viruses and viroid-like elements replicate in fungi. Nat. Commun..

[19] Nuss D.L. (2005). Hypovirulence: Mycoviruses at the fungal–plant interface. Nat. Rev. Microbiol..

[20] Vainio E.J., Rumbou A., Diez J.J., Büttner C. (2024). Forest Tree Virome as a Source of Tree Diseases and Biological Control Agents. Curr. For. Rep..

[21] Nuss D.L. (1992). Biological control of chestnut blight: An example of virus-mediated attenuation of fungal pathogenesis. Microbiol. Rev..

[22] Anagnostakis S.L. (1982). Biological control of chestnut blight. Science.

[23] Yu X., Li B., Fu Y., Xie J., Cheng J., Ghabrial S.A., Li G., Yi X., Jiang D. (2013). Extracellular transmission of a DNA mycovirus and its use as a natural fungicide. Proc. Natl. Acad. Sci. USA.

[24] Sato Y., Suzuki N. (2023). Continued mycovirus discovery expanding our understanding of virus lifestyles, symptom expression, and host defense. Curr. Opin. Microbiol..

[25] Mowna Sundari T., Alwin Prem Anand A., Jenifer P., Shenbagarathai R. (2018). Bioprospection of *Basidiomycetes* and molecular phylogenetic analysis using internal transcribed spacer (ITS) and 5.8S rRNA gene sequence. Sci. Rep..

[26] Mu F., Li B., Cheng S., Jia J., Jiang D., Fu Y., Cheng J., Lin Y., Chen T., Xie J. (2021). Nine viruses from eight lineages exhibiting new evolutionary modes that co-infect a hypovirulent phytopathogenic fungus. PLoS Pathog..

[27] Möller E.M., Bahnweg G., Sandermann H., Geiger H.H. (1992). A simple and efficient protocol for isolation of high molecular weight DNA from filamentous fungi, fruit bodies, and infected plant tissues. Nucleic Acids Res..

[28] Deng Y., Zhou K., Wu M., Zhang J., Yang L., Chen W., Li G. (2022). Viral cross-class transmission results in disease of a phytopathogenic fungus. ISME J..

[29] Nurk S., Meleshko D., Korobeynikov A., Pevzner P.A. (2017). meta-SPAdes: A new versatile metagenomic assembler. Genome Res..

[30] Buchfink B., Xie C., Huson D.H. (2015). Fast and sensitive protein alignment using DIAMOND. Nat. Methods.

[31] Sun A., Zhao L., Sun Y., Chen Y., Li C., Dong W., Yang G. (2023). Horizontal and Vertical Transmission of a Mycovirus Closely Related to the Partitivirus RhsV717 That Confers Hypovirulence in *Rhizoctonia solani*. Viruses.

[32] Edgar R.C. (2004). MUSCLE: Multiple sequence alignment with high accuracy and high throughput. Nucleic Acids Res..

[33] Patro R., Duggal G., Love M.I., Irizarry R.A., Kingsford C. (2017). Salmon provides fast and bias-aware quantification of transcript expression. Nat. Methods.

[34] Wille M., Harvey E., Shi M., Gonzalez-Acuña D., Holmes E.C., Hurt A.C. (2020). Sustained RNA virome diversity in Antarctic penguins and their ticks. ISME J..

[35] Wille M., Shi M., Klaassen M., Hurt A.C., Holmes E.C. (2019). Virome heterogeneity and connectivity in waterfowl and shorebird communities. ISME J..

[36] Oksanen J., Guillaume B.F., Friendly M., Kindt R., Legendre P., McGlinn D., O’Hara R.B., Solymos P., Stevens M.H.H., Szoecs E. Vegan: Community Ecology Package. R Package 2022, Version 2.6-2. https://CRAN.R-project.org/package=vegan.

[37] O’Brien H.E., Parrent J.L., Jackson J.A., Moncalvo J.M., Vilgalys R. (2005). Fungal community analysis by large-scale sequencing of environmental samples. Appl. Environ. Microbiol..

[38] Sahin E., Akata I. (2018). Viruses infecting macrofungi. Virus Dis..

[39] Vainio E.J. (2019). Mitoviruses in the conifer root rot pathogens *Heterobasidion annosum* and *H. parviporum*. Virus Res..

[40] Ohkit S., Lee Y., Nguyen Q., Ikeda K., Suzukib N., Nakayashiki H. (2019). Three ourmia-like viruses and their associated RNAs in *Pyricularia oryzae*. Virology.

[41] Xu Z.Y., Wu S.S., Liu L.J., Cheng J.S., Fu Y.P., Jiang D.H., Xie J.T. (2015). A mitovirus related to plant mitochondrial gene confers hypovirulence on the phytopathogenic fungus *Sclerotinia sclerotiorum*. Virus Res..

[42] Hong Y., Cole T.E., Brasier C.M., Buck K.W. (1998). Evolutionary relationships among putative RNA-dependent RNA polymerases encoded by a mitochondrial virus-like RNA in the Dutch elm disease fungus, *Ophiostoma novoulmi*, by other viruses and virus-like RNAs and by the Arabidopsis mitochondrial genome. Virology.

[43] Polashock J.J., Hillman B.I. (1994). A small mitochondrial double-stranded (ds) RNA element associated with a hypovirulent strain of the chestnut blight fungus and ancestrally related to yeast cytoplasmic T and W dsRNAs. Proc. Natl. Acad. Sci. USA.

[44] Hong Y., Dover S.L., Cole T.E., Brasier C.M., Buck K.W. (1999). Multiple mitochondrial viruses in an isolate of the Dutch elm disease fungus *Ophiostoma novoulmi*. Virology.

[45] Martelli G.P., Adams M.J., Kreuze J.F., Dolja V.V. (2007). Family *Flexiviridae*: A case study in virion and genome plasticity. Annu. Rev. Phytopathol..

[46] Hamid M.R., Xie J., Wu S., Maria S.K., Zheng D., Assane Hamidou A., Wang Q., Cheng J., Fu Y., Jiang D. (2018). A Novel deltaflexivirus that infects the plant fungal pathogen, *Sclerotinia sclerotiorum*, can be transmitted among host vegetative incompatible strains. Viruses.

[47] Li K.F., Zheng D., Cheng J.S., Chen T., Fu Y.P., Jiang D.H., Xie J.T. (2016). Characterization of a novel *Sclerotinia sclerotiorum* RNA virus as the prototype of a new proposed family within the order *Tymovirales*. Virus Res..

[48] Sutela S., Forgia M., Vainio E.J., Chiapello M., Daghino S., Vallino M., Martino E., Girlanda M., Perotto S., Turina M. (2020). The virome from a collection of endomycorrhizal fungi reveals new viral taxa with unprecedented genome organization. Virus Evol..

[49] Forgia M., Isgandarli E., Aghayeva D.N., Huseynova I., Turina M. (2021). Virome characterization of Cryphonectria parasitica isolates from Azerbaijan unveiled a new mymonavirus and a putative new RNA virus unrelated to described viral sequences. Virology.

[50] Linnakoski R., Sutela S., Coetzee M.P.A., Duong T.A., Pavlov I.N., Litovka Y.A., Hantula J., Wingfield B.D., Vainio E.J. (2021). *Armillaria* root rot fungi host single-stranded RNA viruses. Sci. Rep..

[51] Sutela S., Piri T., Vainio E.J. (2021). Discovery and community dynamics of novel ssRNA mycoviruses in the conifer pathogen *Heterobasidion parviporum*. Front. Microbiol..

[52] Tonka T., Walterová L., Čurn V. (2022). Development of RT-PCR for rapid detection of ssRNA ambi-like mycovirus in a root rot fungus (*Armillaria* spp.). Acta Virol..

[53] Melzer M.S., Ikeda S.S., Boland G.J. (2002). Interspecific transmission of double-stranded RNA and hypovirulence from *Sclerotinia sclerotiorum* to *S. minor*. Phytopathology.

[54] Coenen A., Kevei F., Hoekstra R.F. (1997). Factors affecting the spread of double-stranded RNA viruses in *Aspergillus nidulans*. Genet. Res..

[55] Liu Y.C., Linder-Basso D., Hillman B.I., Kaneko S., Milgroom M.G. (2003). Evidence for inter-species transmission of viruses in natural populations of filamentous fungi in the genus *Cryphonectria*. Mol. Ecol..

[56] Shahi S., Eusebio-Cope A., Kondo H., Hillman B.I., Suzuki N. (2019). Investigation of host range of and host defense against a mitochondrially replicating mitovirus. J. Virol..

[57] Wu T., Mao H., Hai D., Cheng J., Fu Y., Lin Y., Jiang D., Xie J. (2023). Molecular characterization of a novel fungal alphaflexivirus reveals potential inter-species horizontal gene transfer. Virus Res..

