# Supplementary material for: Comparative Analysis of Viromes Identified in Multiple Macrofungi
Source: Viruses. 2024 Apr 12;16(4):597. doi: 10.3390/v16040597 (PMC11054281; doi:10.3390/v16040597)
Supplement: Supplementary file 1 [file viruses-16-00597-s001.zip › viruses-2929355-supplementary.pdf]

Supplementary Materials Figures

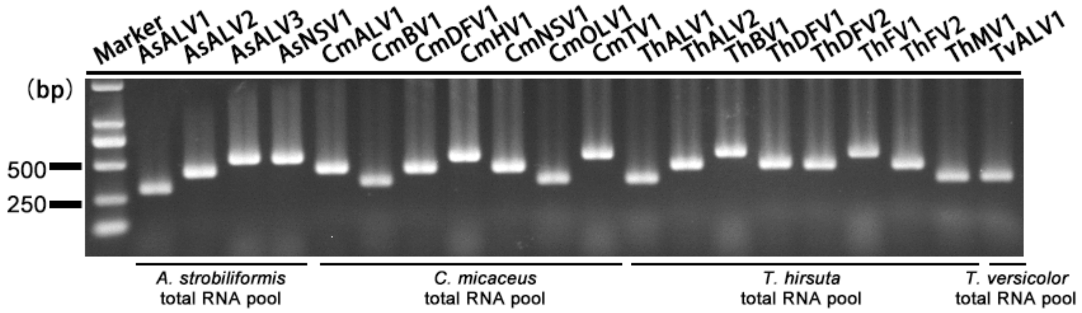

Figure S1: PCR confirmation of mycovirus contigs in four total RNA pools of macrofungi strains (including *Amanita strobiliformis*, *Coprinellus micaceus*, *Trametes hirsuta*, and *Trametes versicolor*, respectively). The viral primers were designed according to the contig sequences, and the primers used are listed in Table S3.

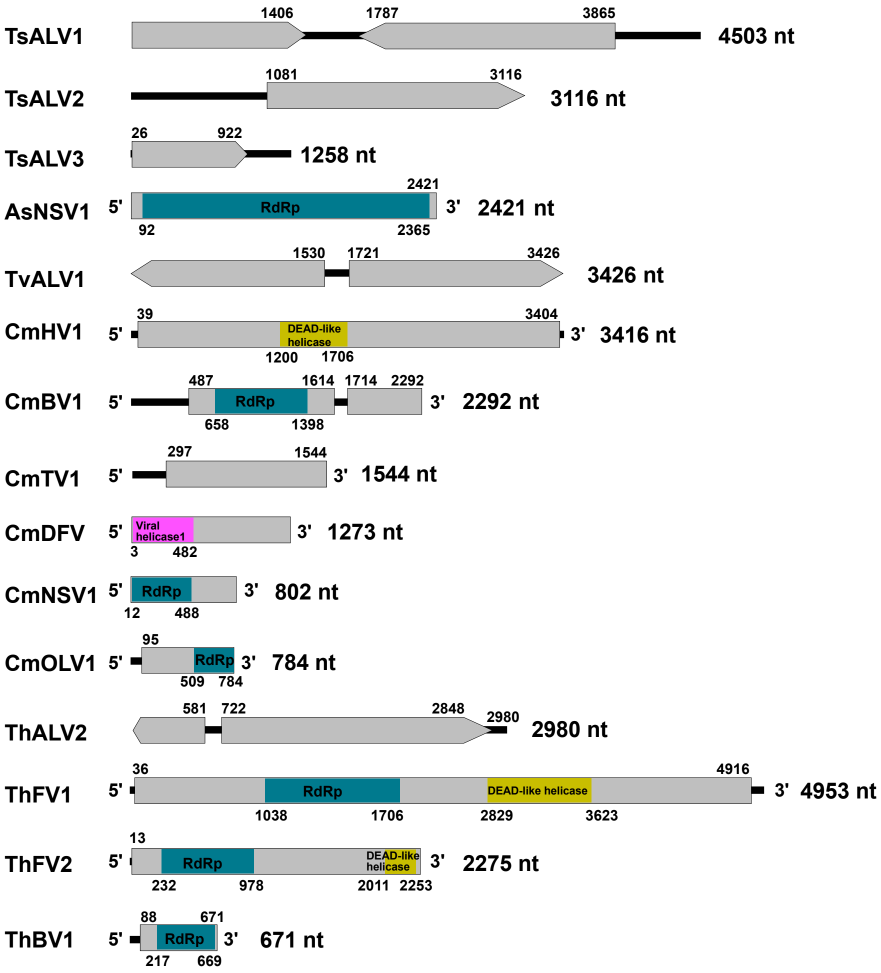

Figure S2: A schematic diagram shows the structure of other viral sequences. The conserved domains include RNA-dependent RNA polymerase (RdRp) and viral helicase (Hel), which are indicated on their fragments.

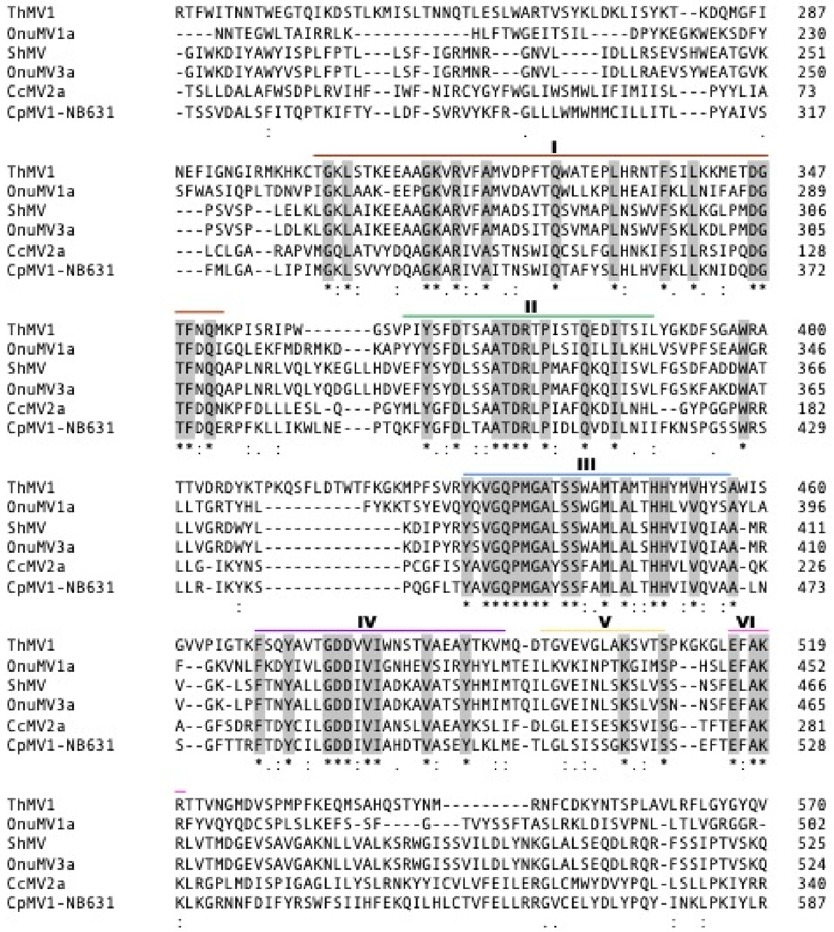

Figure S3: Conserved amino acid sequence motifs of the putative RNA-dependent RNA polymerases of Trametes hirsuta mitovirus 1 (ThMV1), Cryphonectria cubensis mitovirus 2a (CcMV2a), Ophiostoma mitovirus 1a (OnuMV1a), Sclerotinia homoeocarpa mitovirus (ShMV), Ophiostoma mitovirus 3a (OnuMV3a), and Cryphonectria parasitica mitovirus 1-NB631 (CpMV1-NB631). "*" indicates identical amino acid residues; and "." indicates low chemically similar amino acid residues.

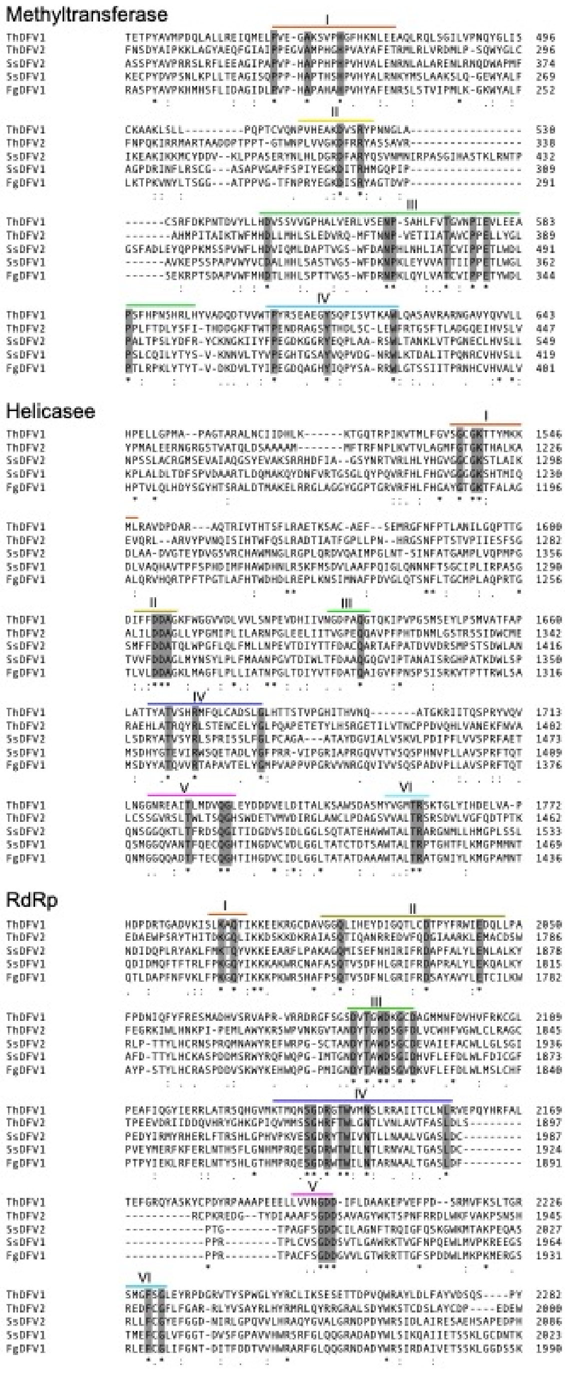

Figure S4: Multiple alignment of amino acid sequences of conserved domains including methyltransferase (Mtr), viral RNA Helicase (Hel), and RNA-dependent RNA polymerase (RdRp) domains of Trametes hirsuta deltaflexivirus 1 (ThDFV1), ThDFV2, Sclerotinia sclerotiorum deltaflexivirus 1 (SsDFV1), Sclerotinia sclerotiorum deltaflexivirus 2 (SsDFV2), and Fusarium graminearum deltaflexivirus 1 (FgDFV1). “*” indicates identical amino acid residues, and “.” indicates low chemically similar amino acid residues.

Supplementary Materials Tables

Table S1. Macrofungi strains from China used in this study

**Sample name**	**Collected data**	**Location**	**Species**	**Total RNA pools**
BFS-1	2021	Shizi Mountain (30° 27' N, 114° 20' E), Huazhong Agricultural University, Wuhan City, Hubei Province, China	*Trametes versicolor*	sequencing library #1
BFS-2	2021		*Trametes versicolor*	
BFS-3	2021		*Trametes versicolor*	
BFS-4	2021		*Trametes versicolor*	
BFS-5	2021		*Trametes versicolor*	
BFS-6	2021		*Trametes versicolor*	
BFS-7	2021		*Trametes versicolor*	
BFS-8	2021		*Trametes versicolor*	
BFS-9	2021		*Trametes versicolor*	
BFS-10	2021		*Trametes versicolor*	
BFS-11	2021		*Trametes versicolor*	
BFS-12	2021		*Trametes versicolor*	
BFS-13	2021		*Trametes versicolor*	
BFS-14	2021		*Trametes versicolor*	
BFS-15	2021		*Trametes versicolor*	
d-1	2021	Shizi Mountain, Huazhong Agricultural University, Wuhan City, Hubei Province, China	*Trametes hirsuta*	sequencing library #2
d-2	2021		*Trametes hirsuta*	
d-3	2021		*Trametes hirsuta*	
d-4	2021		*Trametes hirsuta*	
d-5	2021		*Trametes hirsuta*	
d-6	2021		*Trametes hirsuta*	
d-7	2021		*Trametes hirsuta*	
d-8	2021		*Trametes hirsuta*	
d-9	2021		*Trametes hirsuta*	
d-10	2021		*Trametes hirsuta*	
d-11	2021		*Trametes hirsute*	
d-12	2021		*Trametes hirsuta*	

d-13	2021		*Trametes hirsuta*	
d-14	2021		*Trametes hirsuta*	
d-15	2021		*Trametes hirsuta*	
d-16	2021		*Trametes hirsuta*	
d-17	2021		*Trametes hirsuta*	
d-18	2021		*Trametes hirsuta*	
d-19	2021		*Trametes hirsuta*	
d-20	2021		*Trametes hirsuta*	
DS-1	2021	Shizi Mountain, Huazhong Agricultural University, Wuhan City, Hubei Province, China	*Amanita strobiliformis*	sequencing library #3
DS-2	2021		*Amanita strobiliformis*	
DS-3	2021		*Amanita strobiliformis*	
DS-4	2021		*Amanita strobiliformis*	
DS-5	2021		*Amanita strobiliformis*	
DS-6	2021		*Amanita strobiliformis*	
DS-7	2021		*Amanita strobiliformis*	
DS-8	2021		*Amanita strobiliformis*	
DS-9	2021		*Amanita strobiliformis*	
DS-10	2021		*Amanita strobiliformis*	
DS-11	2021		*Amanita strobiliformis*	
DS-12	2021		*Amanita strobiliformis*	
DS-13	2021		*Amanita strobiliformis*	
DS-14	2021		*Amanita strobiliformis*	
DS-15	2021		*Amanita strobiliformis*	
G-1	2021	Shizi Mountain, Huazhong Agricultural University, Wuhan Province, Hubei Province, China	*Coprinellus micaceus*	sequencing library #4
G-2	2021		*Coprinellus micaceus*	
G-3	2021		*Coprinellus micaceus*	
G-4	2021		*Coprinellus micaceus*	
G-5	2021		*Coprinellus micaceus*	
G-6	2021		*Coprinellus micaceus*	
G-7	2021		*Coprinellus micaceus*	
G-8	2021		*Coprinellus micaceus*	
G-9	2021		*Coprinellus micaceus*	

G-10	2021		*Coprinellus micaceus*	
G-11	2021		*Coprinellus micaceus*	
G-12	2021		*Coprinellus micaceus*	
G-13	2021		*Coprinellus micaceus*	
G-14	2021		*Coprinellus micaceus*	
G-15	2021		*Coprinellus micaceus*	
G-16	2021		*Coprinellus micaceus*	
G-17	2021		*Coprinellus micaceus*	
G-18	2021		*Coprinellus micaceus*	
G-19	2021		*Coprinellus micaceus*	
G-20	2021		*Coprinellus micaceus*	
G-21	2021		*Coprinellus micaceus*	
G-22	2021		*Coprinellus micaceus*	
G-23	2021		*Coprinellus micaceus*	
G-24	2021		*Coprinellus micaceus*	
G-25	2021		*Coprinellus micaceus*	
G-26	2021		*Coprinellus micaceus*	
G-27	2021		*Coprinellus micaceus*	
G-28	2021		*Coprinellus micaceus*	
G-29	2021		*Coprinellus micaceus*	
G-30	2021		*Coprinellus micaceus*	
G-31	2021		*Coprinellus micaceus*	
G-32	2021		*Coprinellus micaceus*	
G-33	2021		*Coprinellus micaceus*	
G-34	2021		*Coprinellus micaceus*	
G-35	2021		*Coprinellus micaceus*	
G-36	2021		*Coprinellus micaceus*	
G-37	2021		*Coprinellus micaceus*	
G-38	2021		*Coprinellus micaceus*	
G-39	2021		*Coprinellus micaceus*	
G-40	2021		*Coprinellus micaceus*	

Table S2 The blastp, PCR confirmation and reads of mycovirus contigs in four species of macrofungi

Short name (Viral contigs)	Viral reads	Host strains	Best match	E-values	Query cover
AsALV1	17777	DS-1, DS-3, DS-4, DS-5, DS-9, DS-10	Phlebiopsis gigantea ambi-like virus 1	2e-104	36%
AsALV2	9839	DS-1, DS-2, DS-8, DS-14	Rhizoctonia solani ambivirus 1	3e-91	56%
AsALV3	4353	DS-6, DS-15	Tulasnella ambivirus 2	2e-14	63%
AsNSV1	22938	DS-1, DS-2, DS-4, DS-5, DS-14	Soybean leaf-associated negative-stranded RNA virus 4	3e-116	96%
CmALV1	958	G-2, G-20	Tulasnella ambivirus 5	4e-123	37%
CmBV1	288	G-3	Sclerotinia sclerotiorum barnavirus 1	8e-48	45%
CmDFV1	96	G-1	Pestalotiopsis deltaflexivirus 1	5e-57	54%
CmHV1	357	G-2, G-15, G-35	Rhizoctonia cerealis hypovirus	5e-173	74%
CmNSV1	50	G-36	Phytophthora condilina negative stranded RNA virus 2	3e-24	62%
CmOLV1	23	G-11	Armillaria mellea ourmia-like virus 1	2e-61	99%
CmTV1	110	G-14	Rhizoctonia solani toti-like virus 1	5e-144	98%
ThALV1	1755	d-5, d-8, d-16, d-17	Fusarium graminearum ambivirus 1	5e-82	44%
ThALV2	345	d-1, d-3	Downy mildew lesion associated ambivirus 2	9e-59	63%
ThBV1	11	d-1	Bemisia tabaci beny-like virus 3	1e-41	99%
ThDFV1	7023	d-1, d-2, d-3, d-16	Lentinula edodes deltaflexivirus 1	0.0	75%
ThDFV2	3494	d-10, d-13, d-17	Cat Tien Macrotermes Deltaflexi-like virus	7e-106	32%
ThFV1	414	d-3, d-9	Phlebiopsis gigantea fusarivirus 1	0.0	70%
ThFV2	221	d-7	Sclerotium rolfsii fusarivirus 2	0.0	99%
ThMV1	7199	d-1, d-2, d-4, d-5, d-7, d-13, d-16, d-20	Lentinula edodes mitovirus 1	0.0	57%
TvALV1	3201	BFS-2, BFS-3, BFS-14	Heterobasidion ambi-like virus 15	2e-94	46%

Table S3 Primers used to detect viral contigs

Name of primers	Sequence from 5' to 3'	Lengths of PCR products (bp)
AsALV1-f	GAGAGAAAGAGCCATTGTG	300
AsALV1-r	GCCTATGGGGCGGTGTATT	
AsALV2-f	GACGCATGACTGCTTGTCCCG	400
AsALV2-r	ATGCAAGTTGATACTACCGC	
AsALV3-f	GGGTCAGCCATTTGTGGCTCC	510
AsALV3-r	CACAACATTTTGCGCTGATTGG	
ASNSV1-f	GTGCCTTGATACTCAAGCACCG	510
ASNSV1-r	CGGAGAACGGACCTGACTTCACG	
CmALV1-f	TGGTCATCTCGGGCTATGGGAA	410
CmALV1-r	GAGGAACTCGAGCGTCAGGCTA	
CmBV1-f	TACCCTCGAAACAAGAACCTGC	320
CmBV1-r	AGACGCTGCCTCTCGGTTCTCTC	
CmDFV1-f	ATGCGCGGAAGACGATGAGT	410
CmDFV1-r	GGCAAGACGACGAAGATATTGGAGAT	
CmHV1-f	TGTGTCTTTTAGAGAGTGGTTAA	511
CmHV1-r	GCTCTATCTATGGCTCGCATGA	
CmNSV1-f	CTGATGATAAAAATGAGTTATTC	413
CmNSV1-r	GTGAGGTGAGTCTCTGAAACCA	
CmOLV1-f	CCAATAAGGAGCTAGCGAGAA	338
CmOLV1-r	GCAACCCTTCCCTGAGTTTG	
CmTV1-f	CATTGAGTGGATGAAATGGCATG	527
CmTV1-r	CTGCTTGTTTACTGGGCTGTAGA	
ThALV1-f	ACAACTCGTAGGCAACCTCC	338
ThALV1-r	TAAATGATAAAGAGCAACACA	
ThALV2-f	CTCCGACCGCTCGATGAGG	455
ThALV2-r	TCTGACAACCACACTCCAACCAC	
ThBV1-f	GGTTCGTCAGGCAGAGGGCC	526
ThBV1-r	GATGGACCTCGGACACGATA	
ThDFV1-f	GCAGAAGAGGTCGAACAACGG	455
ThDFV1-r	GAGTGACGCGAGCGGAGCCTGG	
ThDFV2-f	GCAGCTTTCTGCTTGCGTAC	454
ThDFV2-r	CGGTGAGCAGTGTCCGAAAACAT	
ThFV1-f	AGGGCTGGTTCTTGCTTGGGTA	546
ThFV1-r	CCTCTTTAGAGGCAGTGGGTA	
ThFV2-f	TGCTTTTTGATAGATGCAAACTA	506
ThFV2-r	TTGACCGGGTCGTGATAGACAA	
ThMV1-f	AAACAATAAGACCACTAGAATCT	329
ThMV1-r	GATTACTCTAACCTCTATTATTTC	
TvALV1-f	TGCGGAAAGTGTGGATTCAATG	330
TvALV1-r	TACAACTCACACGATGATGCGCT	
